# Supplementary material for: Intravital quantification reveals dynamic calcium concentration changes across B cell differentiation stages
Source: eLife. 2021 Mar 22;10:e56020. doi: 10.7554/eLife.56020 (PMC8060033; doi:10.7554/eLife.56020)
Supplement: Source code 1. [file elife-56020-code1.docx.zip › Sourcecode.docx]

Source code:

print "ready"

#import sys

#sys.setrecursionlimit(5000)

import numpy as np

import math

from matplotlib.backends.backend_tkagg import FigureCanvasTkAgg

from matplotlib.figure import Figure

from PIL import Image

from scipy.ndimage import gaussian_filter

import Tkinter as tk

import tkFileDialog

import os

print "steady"

np.warnings.filterwarnings('ignore')

###############

## -- GUI -- ##

###############

## ++ choose file ++ ##

root1 = tk.Tk()

root1.filename = tkFileDialog.askopenfilename()

filename = root1.filename

#print filename # whole path & *.tif

directory = os.path.split(filename)[0]

directory = str (directory)

#print directory # whole path without *.tif

baseFolder = os.path.abspath(os.path.join(directory, os.pardir))

rawdataFolder = os.path.basename(directory) # no path only name of previous file

stopFolder = rawdataFolder.find("raw")

folder = rawdataFolder[0:stopFolder-1]

## ++ choose 2PM & binning ++ ##

v = tk.IntVar()

v.set(1)

options = [

("old 2PM + binning1 [153]",153),

("old 2PM + binning2 [76]", 76),

("new 2PM + binning1 [455]",455),

("new 2PM + binning2 [227]",227)

]

def ShowChoice():

global timesteps # == number of images in time stack

timesteps = v.get()

for txt, val in options:

tk.Radiobutton(root1,

text=txt,

padx = 20,

variable=v,

command=ShowChoice,

value=val).pack(anchor=tk.W)

button = tk.Button(root1,text="OK", fg="red", command=root1.destroy)

button.pack(side="bottom")

root1.mainloop()

print " ", timesteps, "timesteps"

### ++ tip sigma & offset ++ ##

root2 = tk.Tk()

sigma = 2

offset = 10

thresh = 0

def show_entry_fields():

global sigma, offset, thresh

sigma = e1.get()

sigma = int(sigma)

offset = e2.get()

offset = int(offset)

thresh = e3.get()

thresh = int(thresh)

tk.Label(root2, text="gaussian blur: sigma = [2]").grid(row = 0)

tk.Label(root2, text="number of steps used for offset [10]:").grid(row = 1)

tk.Label(root2, text="threshold - pixelvalue in SUM-image [0]:").grid(row = 2)

e1 = tk.Entry(root2)

e2 = tk.Entry(root2)

e3 = tk.Entry(root2)

e1.grid(row = 0, column = 1)

e2.grid(row = 1, column = 1)

e3.grid(row = 2, column = 1)

tk.Button(root2, text='use', command=show_entry_fields).grid(row=3, column=1, sticky=tk.W, pady=4)

tk.Button(root2, text='OK', fg="red", command=root2.destroy).grid(row=3, column=0, sticky=tk.W, pady=4)

root2.mainloop()

print " sigma = ", sigma

print " offset = ", offset

print " threshold = ", thresh

print "go "

### ++ choose running variable ++ ##

root4 = tk.Tk()

tk.Label(root4, text = "Which part in filename is running?").grid(row=0, column=0, columnspan=3, sticky=tk.W, pady=4)

substring = [["none", 0, 0],

["Time Time", 5, 8],

["Axis0000", 18, 5],

["xyz-Table Upright Z", 18, 5],

["C00", 0, 3]]

def runV():

global AA

AA = [var1.get(), var2.get(), var3.get(), var4.get(), var5.get()]

AA = np.asarray([index for index, value in enumerate(AA) if value == 1])

print ""

print "running variables:"

for ii in range(len(AA)): print " ", substring[AA[ii]][0]

tauName = ""

realName = ""

imagName = ""

def imaris_yes():

global tauName, realName, imagName, AA, tausi

tauName = "Ch222"

realName = "Ch000"

imagName = "Ch111"

tausi = 1000

print "for Imaris"

runV()

def imaris_no():

global tauName, realName, imagName, tausi

tauName = "tauImage"

realName = "real"

imagName = "imag"

tausi = 1

runV()

var1 = tk.IntVar()

tk.Checkbutton(root4, text=substring[0][0], variable=var1).grid(row=1, sticky=tk.W)

var2 = tk.IntVar()

tk.Checkbutton(root4, text=substring[1][0], variable=var2).grid(row=2, sticky=tk.W)

var3 = tk.IntVar()

tk.Checkbutton(root4, text=substring[2][0], variable=var3).grid(row=3, sticky=tk.W)

var4 = tk.IntVar()

tk.Checkbutton(root4, text=substring[3][0], variable=var4).grid(row=4, sticky=tk.W)

var5 = tk.IntVar()

tk.Checkbutton(root4, text=substring[4][0], variable=var5).grid(row=5, sticky=tk.W)

tk.Label(root4, text = "Do you plan to use IMARIS?").grid(row=8, columnspan=3, sticky=tk.W, pady=4)

tk.Button(root4, text='yes', command=imaris_yes).grid(row=9, column = 0, sticky=tk.W, pady=4)

tk.Button(root4, text='no', command=imaris_no).grid(row=9, column = 1, sticky=tk.W, pady=4)

tk.Button(root4, text='Ok', fg="red", command=root4.destroy).grid(row=9, column = 2, sticky=tk.W, pady=4)

tk.mainloop()

fq = 80E6 # modulation frequence [Hz]

w = 2*math.pi*fq

def reS(tau):

return 1/(1+(w*tau)**2)

def imS(tau):

return w*tau/(1+(w*tau)**2)

################################

## -- NAD(P)H finger print -- ##

################################

tau_free = 0.450E-9 # free NAD(P)H in ns, middle of free region

tau_MDH = 1.2400E-9 # malate dehydrogenase (NADH bound to)

tau_HADH = 1.360E-9

tau_LDH = 1.600E-9 # lactate dehydrogenase

tau_G6PDH = 2.005E-9 # NADPH

tau_SDH_1 = 2.010E-9 # sorbitol dehydrogenase (NADH)

tau_GAPDH = 2.050E-9 #Glyceraldehyde 3-phosphate dehydrogenase

#tau_GPDH1 = 2.070E-9

tau_IDH = 2.170E-9 # Isocitrate dehydrogenase

tau_SDH_2 = 2.260E-9 # sorbitol dehydrogenase (NADPH)

tau_CTBP1_PDH = 2.470E-9

#tau_PDH = 2.470E-9

tau_iNOS = 2.550E-9

tau_ADH = 2.600E-9 # alcohol dehydrogenase

tau_NOX = 3.650E-9 # NADPH

list_tau = (tau_free, tau_MDH, tau_HADH, tau_LDH, tau_G6PDH, tau_SDH_1, tau_GAPDH, tau_IDH, tau_SDH_2, tau_CTBP1_PDH, tau_iNOS, tau_ADH, tau_NOX)

labels_tau = ('free', 'MDH', 'HADH', 'LDH', 'G6PDH', 'SDH (NADH)', 'GAPDH', 'IDH', 'SDH (NADPH)', 'CTBP1/PDH', 'iNOS', 'ADH', 'NOX')

#colors_tau = ((0,0.5,0.4), (1,1,0), (1,0.6,0), (0,1,1), (0,0.2,0.6), (0.5,0,0.5), (1,0,0), (0,0,0), (1,0,1), (0,0,0.5), (0,1,0), (0,0,1), (0.5,0.5,0), (0.5,0,0), (0,0.5,0))

#colors_tau = colors_tau = ((0.0, 0.0, 1.0), (0.0, 0.518, 0.0), (0.0, 0.675, 0.322), (0.0, 0.914, 0.082), (0.0, 1.0, 0.0), (0.514, 1.0, 0.0), (0.859, 1.0, 0.0), (1.0, 1.0, 0.0), (1.0, 1.0, 0.498), (1.0, 1.0, 0.651), (1.0, 0.957, 0.0), (1.0, 0.714, 0.0), (1.0, 0.553, 0.0), (1.0, 0.0, 0.0))

colors_tau = colors_tau = ((0.0, 0.0, 1.0), (0.0, 0.518, 0.0), (0.0, 0.675, 0.322), (0.0, 0.914, 0.082), (0.0, 1.0, 0.0), (0.514, 1.0, 0.0), (0.859, 1.0, 0.0), (1.0, 1.0, 0.0), (1.0, 1.0, 0.498), (1.0, 0.957, 0.0), (1.0, 0.714, 0.0), (1.0, 0.553, 0.0), (1.0, 0.0, 0.0))

def fingerprint():

for num in range(0,len(list_tau)):

ax.plot(reS(list_tau[num]), imS(list_tau[num]), marker='o', markersize=8, color=colors_tau[num], linestyle='none', label=labels_tau[num])

#####################################

## -- define ploting functions -- ##

#####################################

fs = 15 #fontsize plot

def enzymRegion():

## -- enzym region -- ##

rEnzy = []

iEnzy = []

for x in range(77, 108):

rEnzy.append((1+math.cos(math.radians(x)))/2)

iEnzy.append((math.sin(math.radians(x)))/2)

ax.plot(rEnzy, iEnzy, 'k-', linewidth=5)

def NADPHscale():

## -- free -- ##

ax.plot(reS(tau_free), imS(tau_free), 'ko')

ax.text(reS(tau_free), imS(tau_free), "free", va = 'bottom', ha = 'left', rotation = 45, fontsize=fs)

## -- meta. enzymes -- ##

tau_enzyM = 2026E-12 # middle of enzym region

ax.text(reS(tau_enzyM)-0.11, imS(tau_enzyM)+0.02, "meta. enzymes", va = 'bottom', ha = 'left', fontsize=fs)

## -- NOX region -- ##

tau_oxi = 3650E-12 # s; NAD(P)H oxidase

ax.plot(reS(tau_oxi), imS(tau_oxi), 'ko')

ax.text(reS(tau_oxi)-0.01, imS(tau_oxi)+0.01, "NOX", va = 'bottom', ha = 'left', rotation = 45, fontsize=fs)

ax.plot()

def FRETscale_CertNL():

tau_quen = 693E-12 # CerT-NL; Rinnenthal et al, 2013

tau_unquen = 2225E-12

ax.plot(reS(tau_quen), imS(tau_quen), 'ko')

ax.text(reS(tau_quen), imS(tau_quen), "quen.", verticalalignment = 'bottom', horizontalalignment = 'left', rotation = 45, fontsize=fs)

ax.plot(reS(tau_unquen), imS(tau_unquen), 'ko')

ax.text(reS(tau_unquen), imS(tau_unquen), "unquen.", verticalalignment = 'bottom', horizontalalignment = 'left', rotation = 45, fontsize=fs)

ax.plot()

def FRETscale_TNXXL():

# tau_quen = 1260E-12 # TN-XXL; Griesbeck et al

# tau_unquen = 2110E-12

# TN-XXL; Griesbeck et al

tau_quen = 735E-12 # tau1 from suppl tab 1 ECFP

tau_inter = 1260E-12 # from text

tau_unquen = 2350E-12 # tau_ave from suppl tab 1 ECFP

ax.plot(reS(tau_quen), imS(tau_quen), 'ko')

ax.text(reS(tau_quen), imS(tau_quen), "quen.", verticalalignment = 'bottom', horizontalalignment = 'left', rotation = 45, fontsize=fs)

ax.plot(reS(tau_inter), imS(tau_inter), 'ko')

ax.text(reS(tau_inter), imS(tau_inter), "inter.", verticalalignment = 'bottom', horizontalalignment = 'left', rotation = 45, fontsize=fs)

ax.plot(reS(tau_unquen), imS(tau_unquen), 'ko')

ax.text(reS(tau_unquen), imS(tau_unquen), "unquen.", verticalalignment = 'bottom', horizontalalignment = 'left', rotation = 45, fontsize=fs)

def tauMark():

# tau_mark = (0.001E-9, 1E-9, 2E-9, 3E-9, 4E-9, 5E-9, 6E-9, 7E-9, 8E-9, 9E-9, 10E-9)

tau_mark = (0.001E-9, 1E-9, 2E-9, 3E-9, 4E-9, 5E-9, 6E-9, 7E-9, 8E-9, 9E-9, 10E-9, 11E-9, 12E-9, 13E-9, 14E-9, 15E-9)

tau_text = ("0ns", "1ns", "2ns", "3ns", "4ns", "5ns", "6ns", "7ns", "8ns", "9ns", "10ns")

for index in range(len(tau_mark)):

ax.plot(reS(tau_mark[index]), imS(tau_mark[index]), marker='o', markersize=7, color='#bebebe', linestyle='none')

#ax.text(reS(tau_mark[index])+0.01, imS(tau_mark[index])+0.001, tau_text[index], verticalalignment = 'bottom', horizontalalignment = 'left', fontsize=12, color='#bebebe')

#ax.plot([0, reS(tau_free)], [0, imS(tau_free)], color=(0,1,0), linestyle='-')

# ax.plot(reS(tau_free), imS(tau_free), 'ko')

# ax.text(reS(tau_free), imS(tau_free), "free", va = 'bottom', ha = 'left', rotation = 45, fontsize=fs)

# ax.plot([reS(tau_free), reS(tau_LDH)], [imS(tau_free), imS(tau_LDH)], color=(0,1,1), linestyle='-')

#

# ax.plot(reS(tau_LDH), imS(tau_LDH), 'co')

# ax.text(reS(tau_LDH)+0.01, imS(tau_LDH)+0.01, "LDH", va = 'bottom', ha = 'left', fontsize=12)

# ax.plot(reS(tau_MDH), imS(tau_MDH), 'ro')

# ax.text(reS(tau_MDH), imS(tau_MDH), "MDH", va = 'bottom', ha = 'left', fontsize=12)

ax.plot()

def layout():

global real, imag, scale

real = []

imag = []

for x in range(0, 180):

real.append((1+math.cos(math.radians(x)))/2)

imag.append((math.sin(math.radians(x)))/2)

ax.plot(real, imag, 'k-')

ax.set_xlim(0, 1)

ax.set_ylim(0, 0.6)

ax.set_xlabel('real', fontsize=fs)

ax.set_ylabel('imaginary', fontsize=fs)

if scale == 1:

NADPHscale()

enzymRegion()

elif scale == 2:

NADPHscale()

fingerprint()

elif scale == 3:

FRETscale_CertNL()

elif scale == 4:

FRETscale_TNXXL()

elif scale == 5:

tauMark()

else: pass

def contour():

global upCL, lowCL, cf, counts1, levels, extent

if n == 0:

upCL = e.get()

upCL = int(upCL)

lowCL = e2.get()

lowCL = int(lowCL)

else:

upCL

lowCL

canvas=FigureCanvasTkAgg(fig,master=root4)

canvas.get_tk_widget().grid(row=6,column=0, columnspan = 4)

ax.clear()

counts1, ybins1, xbins1 = np.histogram2d(eTAU[:,1], eTAU[:,0], bins=80)

extent = [xbins1.min(), xbins1.max(), ybins1.min(), ybins1.max()]

levels = np.arange(lowCL, upCL, 0.01*upCL)

cf = ax.contour(counts1, levels, linewidths = 1, cmap='plasma', extent = extent)

layout()

def dots():

global upCL, lowCL, cf, counts1, levels, extent

if n == 0:

upCL = e.get()

upCL = int(upCL)

lowCL = e2.get()

lowCL = int(lowCL)

else:

upCL

lowCL

canvas=FigureCanvasTkAgg(fig,master=root4)

canvas.get_tk_widget().grid(row=6,column=0, columnspan = 4)

ax.clear()

# if scale == 2:

# ax.plot(eTAU[:,0], eTAU[:,1], color = 'k', marker='.', linestyle='none', markersize=0.7, zorder=-1)

# else:

ax.plot(eTAU[:,0], eTAU[:,1], color = '#130789', marker='.', linestyle='none', markersize=0.7, zorder=-1)

counts1, ybins1, xbins1 = np.histogram2d(eTAU[:,1], eTAU[:,0], bins=80)

extent = [xbins1.min(), xbins1.max(), ybins1.min(), ybins1.max()]

levels = np.arange(100, upCL, 10)

# if scale == 2:

# cf = ax.contourf(counts1, levels, linewidths = 1, cmap='gist_gray', extent = extent)

# else:

cf = ax.contourf(counts1, levels, linewidths = 1, cmap='plasma', extent = extent)

layout()

#########################

## -- image read-in -- ##

#########################

allfiles = [f for f in os.listdir(directory) if os.path.isfile(os.path.join(directory, f))]

for n in range(0,len(allfiles)):

print ""

print "wait, I'm reading stack ", n, "..."

st = allfiles[n]

stop_dateC = st.find("_DC-TCSPC")

# stop_dateC = st.find("_TDC")

if stop_dateC < 0:

dateCells = st[0:-4]

else:

dateCells = st[9:stop_dateC]

if substring[AA[0]][0] == "none":

uStrich = ""

else: uStrich = "_"

crt = ""

for ix in range(len(AA)):

start = st.find(substring[AA[ix]][0]) + substring[AA[ix]][1]

stop = start + substring[AA[ix]][2]

#print st.find(substring[AA[ix]][0]), start, stop, st[start:stop],

crt += "_" + st[start:stop]

print " ", dateCells + crt

print ""

img = Image.open(directory + '/' + allfiles[n])

shape = img.convert('F')

shape = np.asarray(shape)

shape = shape.shape

## -- without mask -- ##

mask = np.ones(shape)

## -- predefined IJ-mask -- ##

##imgM = Image.open("conva_800nm_P0_BB30_woDC_TDC_maskIJ.tif")

##mask = imgM.convert('F')

##mask = np.asarray(mask)

signal = []

signalG = []

meanG = []

mean = []

for k in range(0,timesteps):

img.seek(k)

data = img.convert('F')

signal.append(np.array(data))

g = 1000*(gaussian_filter(data, sigma=sigma)*mask)

signalG.append(np.array(g))

meanG.append(np.average(signalG[k]))

S = np.array(signal)

S = np.sum(S, axis=0)

sumFile = baseFolder + '/' + str(folder) + '_unblurredSUM/'

if not os.path.exists(sumFile):

os.makedirs(sumFile)

img00 = Image.fromarray(S)

img00.save(sumFile + dateCells + crt + uStrich + 'unblurSUM.tif')

N = np.array(signalG[0:offset]) # average of first 10 images in stack as offset --> subtracted

# N = 0

N = np.average(N)

N = np.ones(shape)*N

datalist = (np.array(signalG)-N)*np.greater(np.array(signalG)-N,0)*np.greater(S, thresh) #subtracts noise & negative values = 0 & thresholds

#############################################

## -- sampling time and used datapoints -- ##

#############################################

dt = 12.24E-9/timesteps

t = []

for k in range(0, timesteps):

t.append(k*dt)

A = []

for k in range(meanG.index(max(meanG)), timesteps):

A.append(datalist[k])

##########################

## -- phasor approach -- ##

###########################

Re = []

Im = []

TAU = []

for k in range(len(A)):

Re.append(A[k]*math.cos(w*t[k]))

Im.append(A[k]*math.sin(w*t[k]))

DFTr = (sum(Re)/sum(A))*np.greater(sum(Re),0)

DFTi = (sum(Im)/sum(A))*np.greater(sum(Im),0)

TAU = (1/w)*(DFTi/DFTr)*1E12 # lifetime in [ps]

#####################

## -- tau image -- ## --> tau in ps

#####################

# upper = 15000.0 # upper border of tau

# lower = 10.0 # lower border of tau

tau = TAU#*(np.less(TAU,upper)*np.greater(TAU,lower)*mask)

analyFile = baseFolder + '/' + str(folder) + '_analy_sig' + str(sigma) + '-off' + str(offset) + '-th' + str(thresh)

tauFile = analyFile + '/tauImages/'

if not os.path.exists(tauFile):

os.makedirs(tauFile)

img1 = Image.fromarray(tau) #tauImage

img1.save(tauFile + dateCells + crt + uStrich + tauName + '.tif')

#######################

## -- phasor plot -- ##

#######################

RE = DFTr#*(np.less(TAU,upper)*np.greater(TAU,lower)*mask)

IM = DFTi#*(np.less(TAU,upper)*np.greater(TAU,lower)*mask)

realFile = analyFile + '/real/'

if not os.path.exists(realFile):

os.makedirs(realFile)

real = Image.fromarray(RE*tausi) # real part image

real.save(realFile + dateCells + crt + uStrich + realName + '.tif')

imagFile = analyFile + '/imag/'

if not os.path.exists(imagFile):

os.makedirs(imagFile)

imag = Image.fromarray(IM*tausi) # imag part image

imag.save(imagFile + dateCells + crt + uStrich + imagName + '.tif')

eTAU = [] # removes zeros from matrix & forms matrix to 1D

for y in range (len(RE)):

for x in range (len(RE[0])):

if RE[y][x] != 0 and IM[y][x] != 0 and np.isnan(TAU[y][x]) == False:

eTAU.append((RE[y][x], IM[y][x], x, y, tau[y][x]))

else: pass

eTAU = np.asarray(eTAU)

### -- contoured 2d histogram -- ###

root4=tk.Tk()

fig = Figure(figsize=(5,3))

ax=fig.add_axes([0,0,1,1])

v = tk.IntVar()

v.set(1)

options = [

("NAD(P)H",1),

("NAD(P)H with enzyms",2),

("FRET (CerT-NL)",3),

("FRET (TN-XXL)",4),

("tau 0-20ns",5),

("no scale",6)

]

def ShowChoice():

global scale

scale = v.get()

print scale

def close():

root4.destroy()

if n == 0:

tk.Label(root4, text = "Tipp contour levels in phasorPlot").grid(row=0, column=0, columnspan=4, sticky=tk.W, pady=4)

tk.Label(root4, text = "lower level (e.g. 0): ").grid(row=1, column=0, sticky=tk.W, pady=4)

e2 = tk.Entry(root4, width = 10)

e2.grid(row=1, column=2)

tk.Label(root4, text = "upper level (e.g. 500): ").grid(row=2, column=0, sticky=tk.W, pady=4)

e = tk.Entry(root4, width = 10)

e.grid(row=2, column=2)

tk.Radiobutton(root4, text=options[0][0], variable=v, command=ShowChoice, value=options[0][1]).grid(row = 3, column = 0)

tk.Radiobutton(root4, text=options[1][0], variable=v, command=ShowChoice, value=options[1][1]).grid(row = 3, column = 1)

tk.Radiobutton(root4, text=options[2][0], variable=v, command=ShowChoice, value=options[2][1]).grid(row = 4, column = 0)

tk.Radiobutton(root4, text=options[3][0], variable=v, command=ShowChoice, value=options[3][1]).grid(row = 4, column = 1)

tk.Radiobutton(root4, text=options[4][0], variable=v, command=ShowChoice, value=options[4][1]).grid(row = 5, column = 0)

tk.Radiobutton(root4, text=options[5][0], variable=v, command=ShowChoice, value=options[5][1]).grid(row = 5, column = 1)

tk.Label(root4, text = "Select your preferred plot type:").grid(row=6, column=0, columnspan=5, sticky=tk.W, pady=5)

plotbutton=tk.Button(master=root4, text="contour", command=contour)

plotbutton.grid(row=7, column=1)

plotbutton=tk.Button(master=root4, text="dots", command=dots)

plotbutton.grid(row=7, column=2)

OKbutton=tk.Button(master=root4, text="OK", fg="red", command=root4.destroy)

OKbutton.grid(row=7, column=32)

tk.mainloop()

else:

contour() or dots()

close()

fig.gca().set_aspect('equal', adjustable='box') # scales plot axis

if contour == True:

fig.colorbar(cf, ax=ax, shrink = 0.8)

else: pass

plotFile = analyFile + '/phasorPlots_enzyLUT/'

if not os.path.exists(plotFile):

os.makedirs(plotFile)

# ax.legend(bbox_to_anchor=(0,1.02,1,0.2), loc="lower left", mode="expand", borderaxespad=0, ncol=3, numpoints=1, scatterpoints = 1)

fig.savefig(plotFile + dateCells + crt + uStrich + 'phasorPlot.png', dpi = 300, bbox_inches='tight')#, transparent=True)

img.close()

print ""

print "Finished!!"
