# Supplementary material for: Intravital quantification reveals dynamic calcium concentration changes across B cell differentiation stages
Source: eLife. 2021 Mar 22;10:e56020. doi: 10.7554/eLife.56020 (PMC8060033; doi:10.7554/eLife.56020)
Supplement: Transparent reporting form [file elife-56020-transrepform.docx]

***eLife’s* transparent reporting form**

We encourage authors to provide detailed information *within their submission* to facilitate the interpretation and replication of experiments. Authors can upload supporting documentation to indicate the use of appropriate reporting guidelines for health-related research (see [EQUATOR Network](http://www.equator-network.org/%20)), life science research (see the [BioSharing Information Resource](https://biosharing.org/)), or the [ARRIVE guidelines](http://www.plosbiology.org/article/info:doi/10.1371/journal.pbio.1000412) for reporting work involving animal research. Where applicable, authors should refer to any relevant reporting standards documents in this form.

If you have any questions, please consult our Journal Policies and/or contact us: [editorial@elifesciences.org](mailto:editorial@elifesciences.org).

**Sample-size estimation**

- You should state whether an appropriate sample size was computed when the study was being designed
- You should state the statistical method of sample size computation and any required assumptions
- If no explicit power analysis was used, you should describe how you decided what sample (replicate) size (number) to use

Please outline where this information can be found within the submission (e.g., sections or **figure legends), or explain why this information doesn’t apply to your submission:**

**Flow cytometry**: Sample size considerations were not met, because read-out is qualitatetivly-descriptive; analyzed cell numbers and volumes per experiment were chosen according to the flow cytometry guidelines cited within the methods section. **Confocal characterization** of GECI (responsiveness vs. unresponsiveness): We could not estimate the effect size according to Cohen upfront (Cohen, Lawrence Erlbaum Associates, 1988). Also, we had to take into account the possibility that the addition of ionomycin might only lead to a small effect. Such would correspond to an estimated Cohen coefficient of d=0.35. We also wanted to define a power of 80% and an alpha error of 1% for our experiments. Accordingly, at least 99 cells would have to be analyzed. Numbers were computed using sample size calculation tools freely available online: <https://statistikguru.de/rechner/stichprobengroesse-gepaarter-t-test-berechnen.html> (Hemmerich, W. (2016)). **Receptor stimulation:** After initial testing of receptor responsiveness, we could adjust the effect size coefficient to 0.8, decreasing the required sample size to at least 15, more cells were analyzed where possible. **Histology:** see **flow cytometry. Intravital microscopy:** according to 3R standards for pilot studies and literature on similiar experiments, the number of mice used was kept as low as possible, with the number of cells analyzed sufficient for reliable statistical testing, i.e. n=51 for aforementioned considerations. In addition, longitudinal intravital microscopy provides the unique advantage of aligning a reduction of sample size with an increase in scientific validity, because they allow extracting more information out of a single sample. Taken into account the number of analyzable cells per measurement ranging from tens to hundreds depending on the parameter analyzed, for in vivo experiments at least three, maximum five mice were used. For bulk cell analysis (e.g. exp. on contacts to SCSM) a randomized pool of 1000 objects (segmented cells at any given time point) was chosen for reasons of clarity.

Information on sample size can be found in the figure legends.

**Replicates**

- You should report how often each experiment was performed
- You should include a definition of biological versus technical replication
- The data obtained should be provided and sufficient information should be provided to indicate the number of independent biological and/or technical replicates
- If you encountered any outliers, you should describe how these were handled
- Criteria for exclusion/inclusion of data should be clearly stated
- High-throughput sequence data should be uploaded before submission, with a private link for reviewers provided (these are available from both GEO and ArrayExpress)

Please outline where this information can be found within the submission (e.g., sections or figure legends), or explain why this information doesn’t apply to your submission:

Number of replicates is stated in each figure legend where applicable.

Cells unresponsive to stimulation were excluded from descriptive data on receptor stimulation; for in vivo analysis of cells over time, cells not traceable for at least 20 minutes were excluded. Furthermore, cells with insufficient signal-to-noise ratio were excluded from quantitative calcium measurements.

This and further information can be found in the figure legends, within the methods section (“Intravital and live cell imaging and image analysis”) and the section “Statistical information”.

Data obtained will be made available using a public Dryad upon full submission process.

Information on this can be found in the section “Data availability”.

**Statistical reporting**

- Statistical analysis methods should be described and justified
- Raw data should be presented in figures whenever informative to do so (typically when N per group is less than 10)
- For each experiment, you should identify the statistical tests used, exact values of N, definitions of center, methods of multiple test correction, and dispersion and precision measures (e.g., mean, median, SD, SEM, confidence intervals; and, for the major substantive results, a measure of effect size (e.g., Pearson's r, Cohen's d)
- Report exact p-values wherever possible alongside the summary statistics and 95% confidence intervals. These should be reported for all key questions and not only when the p-value is less than 0.05.

Please outline where this information can be found within the submission (e.g., sections or figure legends), or explain why this information doesn’t apply to your submission:

Information on this can be found in each figure legend, where applicable.

(For large datasets, or papers with a very large number of statistical tests, you may upload a single table file with tests, Ns, etc., with reference to sections in the manuscript.)

**Group allocation**

- Indicate how samples were allocated into experimental groups (in the case of clinical studies, please specify allocation to treatment method); if randomization was used, please also state if restricted randomization was applied
- Indicate if masking was used during group allocation, data collection and/or data analysis

Please outline where this information can be found within the submission (e.g., sections or figure legends), or explain why this information doesn’t apply to your submission:

Does not apply. As a tool for basic biological research, the study focuses on a novel methodology and describes cellular behavior of cells in situ without manipulations. Therefore, no control group or randomization is necessary.

**Additional data files (“source data”)**

- We encourage you to upload relevant additional data files, such as numerical data that are represented as a graph in a figure, or as a summary table
- Where provided, these should be in the most useful format, and they can be uploaded as “Source data” files linked to a main figure or table
- Include model definition files including the full list of parameters used
- Include code used for data analysis (e.g., R, MatLab)
- Avoid stating that data files are “available upon request”

Please indicate the figures or tables for which source data files have been provided:

Source data files will be deposited at <https://datadryad.org> upon full submission.

Python-based code for absolute calcium quantification will be deposited at <https://github.com>
